# Isolation and Genetic Characterization of the Human Relapsing Fever Spirochete *Borrelia persica* from a Dog with Improved Cultivation Techniques

**DOI:** 10.1155/2024/4144123

**Published:** 2024-03-22

**Authors:** Dor Shwartz, Yaarit Nachum-Biala, Bar Ben-Shitrit, Reinhard K. Straubinger, Gad Baneth

**Affiliations:** ^1^Koret School of Veterinary Medicine, Hebrew University of Jerusalem, P.O. Box 12, Rehovot 7610001, Israel; ^2^Bacteriology and Mycology, Institute for Infectious Diseases and Zoonoses, Ludwig-Maximilians-Universität München, Oberschleißheim, Munich, Germany

## Abstract

*Borrelia persica* is the causative agent of tick-borne relapsing fever in Israel and is prevalent in the Eastern Mediterranean basin and parts of Asia. Infection with *B. persica* causes severe illness and potentially life-threatening complications in humans and companion animals. Isolation and *in vitro* cultivation of *B. persica* in culture medium is difficult and there are only two previous isolates of this spirochete. Here, we describe the first cultivation of *B. persica* from an ill dog. Isolation from the dog's blood was performed with the Pettenkofer-LMU *Bp* medium and spirochetes with vibrant motility and cell density of 2 × 10^6^/ml were observed in culture by dark-field microscopy 3 days after sampling. The isolate was passaged every 3–4 days with cell densities reaching up to 3 × 10^7^/ml achieved over 30 passages. Cryopreservation was made at −80°C without any cryoprotectant additive, and successful growth from thawed culture samples was observed up to 3 months after freezing with repeated freeze and thaw cycles. Generation time during the exponential growth phase was 14.6 hr. Genetic characterization by polymerase chain reaction amplification and DNA sequencing of the flagellin, glycerophosphodiester phosphodiesterase, and *16S rRNA* genes indicated that the isolate is a *B. persica* genotype I strain associated with human relapsing fever. The new canine isolate would be helpful for research on the pathogenesis of relapsing fever and the new modifications in cultivation and preservation methods may assist in future studies of relapsing fever by simplifying previously reported *in vitro* methods.

## 1. Introduction

Spirochetes of the relapsing fever (RF) group *Borrelia* are the causative agents of RF in humans and companion animals [1, 2]. Tick-borne relapsing fever (TBRF) is caused by different *Borrelia* species which are mostly transmitted by argasid ticks from the genus *Ornithodoros* with some exceptions such as *Borrelia miyamotoi* transmitted by ixodid ticks [3, 4]. *Borrelia persica* was reported as the causative agent of TBRF in several countries including Iran, Pakistan, China, Uzbekistan, Tajikistan, and Egypt, and it is the only reported endemic RF species in Israel [5–10]. *B. persica* is transmitted by the tick *Ornithodoros tholozani*, whose geographic distribution extends from India and Central Asia to Egypt and largely overlaps the distribution of *B. persica* [11, 12]. Infection with *B. persica* has been reported in various wild and domestic animal species including the domestic dog (*Canis lupus familiaris*), cat (*Felis catus*), European badger (*Meles meles*), striped hyena (*Hyaena hyaena*), golden jackal (*Canis aureus*), red fox (*Vulpes vulpes*), rock hyrax (*Procavia capensis*), and several rodent species including the social vole (*Microtus socialis*), fat sand rat (*Psammomys obesus*), and Cairo spiny mouse (*Acomys cahirinus*) [13–16]. Movement of infected hosts between TBRF endemic and TBRF-free areas poses the risk of introduction of the infection into new countries [10, 17].

In humans, RF is characterized by recurrent fever episodes, which usually correspond to increasing spirochetemia waves, and are accompanied by headache, lethargy, tachycardia, conjunctivitis, hepatomegaly, splenomegaly, pigmenturia, vomiting, myalgia, and arthralgia [2, 8, 18]. RF during pregnancy may lead to abortion or stillbirth [19].

The reported incidence of TBRF in civilians in Israel has declined from 0.35 cases per 100,000 inhabitants in the years 1975–1985 to 0.11/100,000 from 1986 to 2003. Nevertheless, the incidence among Israeli military personnel remained stably high with an annual average of 6.4 cases per 100,000 [20]. The mortality rates of Old World TBRF in untreated human patients are in the ranges of 4%–10% and are largely influenced by coexisting conditions, such as malnutrition, dehydration, and coinfection. Although mortality rates decrease with prompt antibiotic treatment to less than 2%, severe life-threatening complications, such as aseptic meningitis, acute respiratory distress syndrome, and acute neonatal death were reported in human patients infected with *B. persica* [21, 22]. In dogs and cats, RF caused by *B. persica* is associated with clinically evident illness and clinicopathological findings, such as fever, lethargy, anorexia, anemia, and thrombocytopenia, and can be fatal [23]. Clinical disease in pets was reported in infection with other RF species including *B. turicatae*, *B. hermsii*, and *B. hispanica* [24–26].

One of the most challenging aspects of borreliosis research is the ability to successfully cultivate long-term stable cultures of RF species. Some of the RF species are notoriously difficult to cultivate *in vitro* [27, 28]. Early attempts to cultivate *Borrelia* spirochetes were made by inoculating blood from naturally infected subjects into laboratory animals and serial passages of infected blood to other animals [29]. Over the past decades, several cultivation media were developed for cultivating *Borrelia* spp. including the Barbour–Stoenner–Kelly (BSK) medium, modified Kelly–Pettenkofer (MKP), and variations of these media, which made *in vitro* cultivation of many *Borrelia* species possible [30, 31]. Although some RF *Borrelia* spp. have been adapted to BSKII medium, *B. persica* could not be cultured *in vitro* until 2015, when the pathogen was isolated from the blood of a cat diagnosed with this infection in Israel and from ticks collected in a cave where humans were infected in Israel [32]. These strains were successfully isolated using a new modification of the MKP medium, named the Pettenkofer-LMU *Bp* medium (Pett./LMU Bp.). The first isolation and the first two passages of these strains were done into human serum and then transferred into tubes containing Pett./LMU Bp. medium.

Glycerol is used as a cryoprotective additive at different concentrations for conserving and freezing a variety of microorganism including viruses, bacteria (including rickettsia and mycoplasma), yeasts, algae, and protozoa [33]. Nevertheless, although glycerol was found to have a positive cryoprotective effect on diverse microorganisms, it was observed to have a toxic effect on others, with deleterious effect on infectivity, such as in the case of several serovars of the spirochete *Leptospira interrogans*, *Trypanosoma* spp., *Anaplasma phagocytophilum*, and *Plasmodium* spp. [34–38].

In this study, we describe the first isolation and *in vitro* cultivation of *B. persica* from a dog and new modifications to simplify its isolation and cryopreservation.

## 2. Materials and Methods

### 2.1. *B. persica* Strain Collection and Isolation

**Figure 1:**
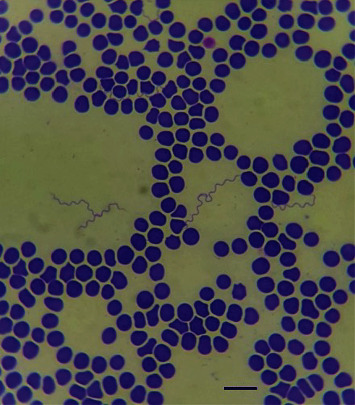
Spirochetemia with *B. persica* in a blood smear from the infected dog; May–Grünwald–Giemsa stain. Scale bar: 10 *μ*m.

Blood was collected in a 5 ml EDTA tube (Sarstedt AG & Co., Numbrecht, Germany) from a sick dog brought for veterinary care in the city of Beer Sheva in Southern Israel. A blood smear, which was made and stained with May–Grünwald–Giemsa by an attending veterinary clinician, showed spirochetemia suspected as infection with a RF *Borrelia* spp. (Figure 1) [23]. One hundred microliters of the blood were transferred to a microfuge tube (SSIbio, Lodi, CA, USA) for DNA extraction. The rest of the blood (∼2 ml) was divided into two microfuge tubes, which were centrifuged for 10 min at 350x *g*. The precipitate was washed three times with sterile phosphate-buffered saline. The supernatant was discarded after the final wash and the precipitate containing spirochetes was mixed with 3 ml of prewarmed Pettenkofer-LMU Bp medium, as previously described [32] in a 5 ml polypropylene tube (Sarstedt AG & Co., Numbrecht, Germany) and incubated at 37°C with no additional CO_2_ supplementation [32].

### 2.2. Molecular Analysis

DNA was extracted from 100 *µ*l of culture and from the dog's blood using a commercial kit (DNeasy Blood and Tissue Kit, Qiagen, Germany) following the manufacturer's protocol. Real-time and conventional polymerase chain reactions (PCR) were performed on DNA extracted from the culture and blood samples by the amplification of three different genes of RF borreliae. An approximately 346-bp fragment of the *flaB* gene was amplified as previously reported for other *Borrelia* species using primers Bfpbu and Bfpcr [39] and an approximately 280 bp fragment of the glycerophosphodiester phosphodiesterase (*GlpQ*) gene, which is specific for RF borreliae, was amplified using primers 510f and 770r [40]. In addition, an approximately 515 bp fragment of the *Borrelia* 16S ribosomal RNA (*16S rRNA*) gene was amplified using primers rec4 and rec9 [41]. Furthermore, to detect possible coinfection with other tick-borne disease agents, real-time, and conventional PCRs were performed on DNA from the dog's blood sample for the detection of *Ehrlichia canis*, *Babesia* spp., and *Hepatozoon* spp. (Table 1).

The *flaB* and *glpQ* real-time PCR protocols were carried out with an initial hold for 3 min at 95°C, followed by 45 cycles of 15 s at 95°C, 30 s at 58°C, and 10 s at 72°C. The melting phase started at 60°C, each step rising by 0.3°C/s, and finishing at 95°C with a hold for 90 s at the first step and 5 s at the subsequent steps. Each reaction was performed in 20 *μ*l reaction volume containing 4 *μ*l of sample DNA, 0.5 *μ*M of each primer, 0.6 *μ*l of Syto9 (Invitrogen, CA, USA), 4.4 *μ*l of ultra-pure water, and 10 *μ*l of DreamTaq PCR Master Mix (Thermo Scientific, Loughborough, UK). All reactions were performed in the Life Technologies StepOnePlus real-time PCR system (Thermo Fisher Scientific, Waltham, MA, USA). The *E. canis* real-time PCR protocol was carried out as previously described [43]. The *flaB* conventional PCR protocol was carried out for 45 cycles consisting of denaturation at 94°C for 30 s, annealing at 56°C for 30 s, and extension at 72°C for 30 s, and there was a final extension step consisting of 5 min at 72°C. The *16S* rRNA conventional PCR protocol was carried out for 35 cycles consisting of denaturation at 96°C for 1 min, annealing at 94°C for 1 min, and extension at 72°C for 1 min, and there was a final extension step consisting of 5 min at 72°C. The *18S rRNA* piroplasmid conventional PCR protocol was carried out, as previously described [44]. Each reaction was performed in 25-*μ*l reaction volume containing 4 *μ*l of sample DNA, 1 *μ*M of each primer, and 19 *μ*l of distilled extra pure water. DNA extracted from 3 ml of *B*. *persica* culture was used as a positive control. All positive PCR products were sequenced from both directions using the respective forward and reverse primers. Sequencing was performed by the BigDye Terminator cycle sequencing chemistry from Applied Biosystems ABI 3700 DNA Analyzer (ABI, Carlsbad, CA, USA) and the ABI Data Collection and Sequence Analysis software at the Center for Genomic Technologies, Hebrew University of Jerusalem, Israel. DNA sequences were aligned using the Molecular Evolutionary Genetics Analysis software MEGA, version 11.013 [45]. Sequences were further compared to the GenBank database using the BLAST algorithm (National Center for Biotechnology Information, Bethesda, USA; http://blast.ncbi.nlm.nih.gov/Blast.cgi). Species-level identification was obtained according to the closest BLAST match when sequences showed identity higher than 99% to the first match GenBank sequences.

### 2.3. Growth Characteristics

The growth dynamics of *B. persica* strain HU-D03 in Pett./LMU *Bp* were evaluated for long-term stability and propagation characteristics. The long-term stability of the isolate was evaluated by inoculation of 100 *µ*l of the culture's first passage (P1) in 3 ml Pett./LMU Bp medium in triplicates and subsequent incubation at 37°C. The cultures were checked every 24–48 hr, and the viable cell density was measured at day 3 or 4, using the improved Neubauer counting chamber (Merck KGaA, Darmstadt, Germany). Only motile spirochetes were included in the counting. When bacterial growth reached cell density of at least 1× 10^6^/ml with highly motile spirochetes, 100 *µ*l of the culture were transferred into 3 ml of fresh medium, and passages were made every 3 or 4 days up to passage 30. Spirochete growth dynamic was studied in repeated reactions (six times) as follows: 100 *µ*l of *B. persica* stock passage (P7) were introduced into 3 ml Pett./LMU Bp medium and incubated at 37°C. The cultures underwent spirochete counts daily for 11 days and analysis of bacterial growth was carried out including cell counts and evaluation of spirochete viability [46]. Generation time was calculated using the equation *G* = *t*/3.3 (log *b*/B), where *G* is generation time, *b* is the number of cells at the end of the exponential phase, B is the number of cells at the beginning of exponential phase, and *t* is the time interval between the beginning and the end of exponential phase [47]. Data on growth dynamics were analyzed using Microsoft Excel Software (Microsoft Corporation 2013).

### 2.4. Culture Maintenance

Four days after the spirochete's inoculation into the culture medium as passage 1 (P1), 100 *µ*l of culture were transferred to 3 ml of prewarmed medium and designated as passage 2 (P2) of *B. persica* strain HU-DO3. Additional passages from live cultures were further made by transferring 100 *µ*l of culture to 3 ml of prewarmed medium every 3–4 days and testing for viability by dark-field microscopy. Cultures of the passages were incubated at 37°C, and a portion of 100 *µ*l of each passage starting from P2 was frozen directly at −80°C without any cryopreserving additive at day 3 of the culture, in sterile 1.5 ml microfuge tubes (Labcon, Langenhagen, Germany). Freezing was carried out following testing for viability when reaching a cell density of 1 × 10^6^ spirochetes/ml or above, possessing high viability as measured by motility speed and intact morphology. The thawing process was made by quick defrosting of the aliquots by warming the tube to body temperature (37°C) in a laboratory water bath and when completely defrosted, immediate transfer of 100 *µ*l into prewarmed medium at 37°C.

## 3. Results

A 10 years old, female spayed boxer dog was admitted to a veterinary clinic due to lethargy. It had a rectal temperature of 39.5°C indicating mild fever. A complete blood count showed mild normocytic normochromic anemia (Hematocrit 34.5%, reference range (RI): 37.3%–61.7%; mean cell volume (MCV) 69.1 fL, RI: 61.6–73.5 fL; mean corpuscular hemoglobin concentration (MCHC) 36.6 g/dl, RI: 32–37.9 g/dl) and severe thrombocytopenia (5,000 per *µ*l; RI: 148,000–484,000 per *µ*l). Blood smear microscopic evaluation showed no red blood cells regeneration, confirmed the thrombocytopenia, and demonstrated infection with spirochetes suspected as RF *Borrelia* spp. (Figure 1).

Serum biochemistry panel showed only a mild elevation in alanine transaminase (ALT) activity (139 U/L; RI: 10–125 U/l). Real-time PCR of blood was positive for the *Borrelia flaB*, *glpQ*, and *16S rRNA* genes, and negative for *E. canis*, *Babesia* and *Hepatozoon* species. Sequencing analysis of the *flaB* and *16S rRNA* genes showed 100% identity to *B. persica* GenBank accession numbers KX258795.1 and KU565880.1 previously amplified from an *O. tholozani* tick and from a cat in Israel, respectively. Furthermore, sequence analysis of the *glpQ*-gene showed 99.6% identity to *B. persica* GenBank accession MH923346.1 previously amplified from an *O. tholozani* in Israel.

**Figure 2:**
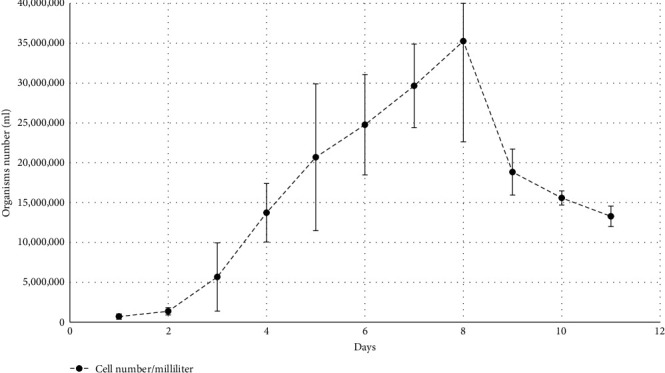
Growth curve of the *B. persica* strain HU-D03 (P9) showing the mean daily cell number/milliliter and standard error.

A cell density of 2 × 10^6^ viable spirochetes per ml was observed by dark-field microscopy 3 days after the initial cultivation of *B. persica* HU-D03 (P1) in Pett./LMU Bp medium which originated from the blood of a spirochetemic febrile dog. Growth behavior dynamics are shown in Figure 2. During the first 4 days, the spirochete motility was intense and the microorganisms presented in ring forms and clear helical shapes. The highest cell densities were measured on day 8, with an average of 3.5 × 10^7^/ml. From day 6, the motility decreased, and the ring formation declined. From day 8, the number of motionless and partially dissolved spirochetes increased gradually.

**Figure 3:**
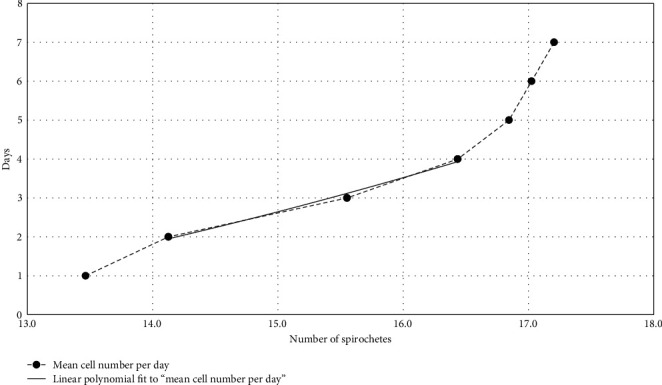
A logarithmic representation of the values of Figure 2. The continuous line follows the values in the exponential phase of the growth.

Generation time was calculated based on the mean cell numbers during the exponential phase, as shown by the logarithmic representation of the spirochete growth (Figure 3). The generation time of *B. persica* strain HU-D03 in Pett./LMU Bp medium was calculated based on two-time intervals in the exponential growth phase: The mean cell number/milliliter at the end of exponential phase (day 4) was 1.4 × 10^7^ and the mean cell number/milliliter at the beginning of exponential phase (day 2) was 1.4 × 10^6^. Based on the equation *G* = *t*/3.3 log(*b*/B), the generation time was calculated as 14.6 hr.

Successful propagation of thawed cryoprotectant-free *B. persica* strain HU-D03 was achieved four times using different passages (P5, P6, P7, and P24). These aliquots were frozen for 2–3 months before thawing. To evaluate the spirochetes' ability to propagate well after prolonged freezing, two additional freeze and thaw cycles of one of the cultures (P5) were made showing that the isolates revived and grew successfully.

**Figure 4:**
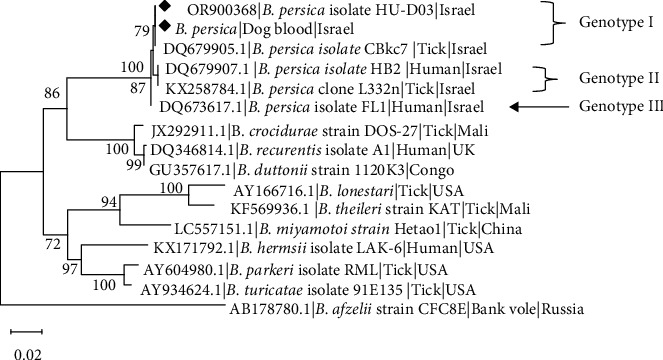
A maximum likelihood phylogram comparing 706-bp DNA fragment sequences of the *flaB* gene from the canine blood and cultured *B. persica* strain included in the study to other *B. persica* sequences and *Borrelia* spp. (GenBank accessions). New sequences derived from this study are marked with black diamonds. Note the devision of *B. persica* genotypes marked in Roman numerals. The GenBank accession numbers, species of infected host, and country of origin are included for each sequence. The Tamura-3-parameter model was used in the construction of this phylogram with bootstrap values deduced from 1,000 replicates, and values higher than 70% are indicated.

Sequencing data were obtained for both blood and culture (P3). For the *Borrelia flaB* gene, 706-bp sequences amplified from the blood and from culture were found 100% identical to each other and 100% identical to *B. persica* (KX258795.1) and deposited in GenBank under accession number OR900368. Phylogenetic analysis based on a 706-bp segment of the *flaB* gene sequence (Figure 4) revealed that both blood and culture isolations of *B. persica* sequences from this study clustered together with a *B. persica* genotype I sequence from an *O. tholozani* tick collected in Israel (GenBank DQ679905.1), and separately from *B. persica* genotypes II (GenBank DQ679907.1) and III (GenBank DQ673617) previously amplified from human patients in Israel [42]. All *B. persica* sequences clustered separately from other Old-World RF *Borrelia* spp. including *B. recurrentis*, *B. duttonii*, and *B. crocidurae*, and from North American RF species including *B. parkeri*, *B. turicatae*, and *B. hermsii* which also clustered separately.

Sequencing data of a 235-bp amplified fragment of the *Borrelia glpQ*-gene which was deposited in GenBank as accession number OR900369 showed that sequences obtained from the blood and culture (P3) were 100% identical to each other and 99.6% identical to *B. persica* (accession number MH923346.1).

**Figure 5:**
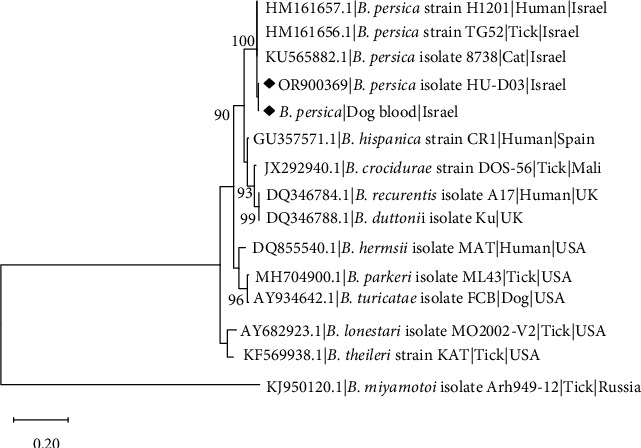
A maximum likelihood phylogram comparing 249-bp DNA fragment sequences of the *glpQ*-gene from the canine blood and cultured *B. persica* strain included in the study to sequences from other *B. persica* and other *Borrelia* spp. (GenBank accessions). New sequences derived from this study are marked with black diamond squares. The GenBank accession numbers, species of infected host, and country of origin are included for each sequence. The Tamura-3-parameter model was used in the construction of this phylogram with bootstrap performed on 1,000 replicates, and values higher than 70% are indicated.

Phylogenetic analysis of the 235-bp *glpQ*-segment (Figure 5) revealed that sequences from this study clustered with other *B. persica* sequences from a human, cat, and an *O. tholozani* tick. As in the *flaB* phylogram, *B. persica glpQ*-sequences clustered separately from Old-World RF *Borrelia* spp. including *B. hispanica*, *B. duttonii*, *B. crocidurae*, and *B. recurrentis*. The Old-World species including *B. persica* also clustered separately from American RF *Borrelia* spp., namely *B. hermsii*, *B. parkeri*, and *B. turicatae*.

**Figure 6:**
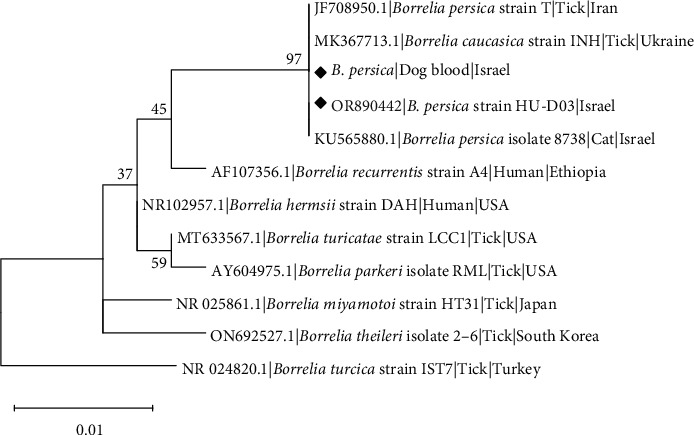
Maximum likelihood phylogram comparing 407-bp DNA fragment sequences of the *Borrelia 16S rRNA* gene from the canine blood and cultured *B. persica* strain included in the study to sequences of other *B. persica* and *Borrelia* species (GenBank accessions). New sequences derived from this study are marked with black diamond squares. The GenBank accession numbers, species of infected host, and country of origin are included for each sequence. The Kimura-2-parameter model was used in the construction of this phylogram with bootstrap performed on 1,000 replicates.

Sequencing data of a 484-bp sequence of the *Borrelia* 16S rRNA-gene obtained from both dog blood and culture (P3) and deposited in GenBank as accession number OR890442 showed that sequences from the blood and culture were 100% identical to each other and 100% identical to *B. persica* (accession number KU565880.1). Phylogenetic analysis of a 407-bp segment of the *Borrelia 16S rRNA*gene (Figure 6) showed that sequences from this study clustered with other *B. persica* sequences from a human, cat, and an *O. tholozani* tick, and also with *B. caucasica* isolated from an *O. verrucosus* tick. *B. persica 16S rRNA* sequences showed similar grouping patterns to the *flaB* and *glpQ* phylograms, as they clustered separately from Old-World and North American RF *Borrelia* spp., including B. *recurrentis*, *B. theileri*, *B. hermsii*, *B. turicatae*, and *B. miyamotoi*.

## 4. Discussion

This study describes the first cultivation of the RF spirochete *B. persica* isolated from a dog. *B. persica* is considered difficult to culture in medium and before 2015, there were no cultivated isolates grown *in vitro* of this spirochete, and it was only possible to pass it in laboratory animals for research purposes [7, 48]. In 2015, *B. persica* was isolated successfully from a cat and an *O. tholozani* tick and these are the only existing isolates of this *Borrelia* spp. until the current canine isolation described herein [32]. Cultivation described previously required the initial use of freshly prepared human serum and revival of the isolates following freezing in glycerol was difficult and often unsuccessful in the author's experience [32]. The current study has found a simplified successful and reliable method for directly freezing and thawing of *B. persica* isolates without using glycerol or other preservatives.

Clinical disease due to RF *Borrelia* spp. has been reported in domestic dogs from Israel and Iran infected with *B. persica*, dogs from North America infected with *B. turicatae* and *B. hermsii*, and dogs from Europe infected with *B. hispanica* [23, 24, 26, 49–51]. Previous reports described the successful isolation and cultivation of the North American RF species, *B. turicatae*, and *B. hermsii*, from spirochetemic dogs. The cultivation method described for *B. turicatae* included direct isolation into a BSKII medium in one report [25]; however, isolation of *B. hermsii* and *B. turicatae* in other reports required two *in-vivo* steps of inoculating laboratory mice (*Mus musculus*) with the dogs' infected blood samples or sera [23, 24].

The growth dynamics of the isolated canine strain of *B. persica* showed similar characteristics to the growth dynamics found for the previous cat and *O. tholozani* tick isolates, with approximately 2 days of lag phase and 3 days of log phase. Furthermore, the maximal cell density was observed on day 8 for all mentioned isolates. However, the cell density reached during growth of the canine isolate was approximately 10 times higher than those reported for the cat and tick isolates [30]. Future studies will be directed to compare the growth dynamics of *B. persica* and other RF *Borrelia* spp. using the same medium and under the same conditions in order to improve the characterization of the growth of the new canine *B. persica* isolate.

The generation time of *Borrelia* spp. varies and range 7–20 hr [52]. The generation time of the isolated canine strain of *B. persica* from this study (14.6 hr) was shorter than previously reported for the cat and tick isolates (19.1 hr), but longer than reported for other RF *Borrelia* spp. such as *B. hermsii* (7 hr) and *B. recurrentis* (8–9 hr) [32, 53–55].

In contrast to the well-established knowledge on the genetic structure, epidemiology, clinical features, and virulence factors of *Borrelia burgdorferi* that belongs to the Lyme disease group, knowledge on some of the RF *Borrelia* group species, and *B. persica* in particular, is lacking and only a few RF species have been comprehensively studied [56–58]. The ability to grow an *in vitro* culture of *B. persica* is of great importance to the study of the pathogenesis of this spirochete. Therefore, the successful methods of isolation, cultivation, and preservation reported in this study may provide an opportunity to study the infection mechanisms of *B. persica* in detail.

Phylogenetic analysis based on *flaB*, *glpQ*-, and *16S rRNA* gene sequences of the isolated canine strain of *B. persica* in this study revealed identity or very close genetic proximity to other *B. persica* sequences reported previously from human patients, ticks, and animals. Furthermore, a close genetic proximity of *B. persica* genotype III to *B. caucasica*, which causes TBRF in Northeastern Europe and the Caucasus area, was observed with particular attention to the *flaB* and *16S rRNA* sequences, as reported previously [59]. Comprehensive genetic studies using techniques such as whole genome sequencing analysis often require isolation and cultivation of the studied species to reach optimal outcomes [59, 60]. The development of reliable isolation and cultivation methods will enable future studies on the genetic and molecular basis of pathogenicity of RF species. Furthermore, it will allow the comparison of RF species, such as *B. persica* to other closely related species such as *B. caucasica*.

Cryopreservation of *in vitro* cultures is of considerable importance in the study of RF spirochetes. Glycerol preservation has been shown to be toxic in some cases. In a previous study, the cryoprotective effect of glycerol on the growth of several serovars of *L. interrogans* was compared to dimethyl sulphoxide (DMSO), which is also used frequently for bacterial preservation. Glycerol was found to have a deleterious effect on bacterial growth 10 times more than DMSO, and all serovars tested including *L. interrogans serovar Canicola*, *L. interrogans serovar Hardjo*, *L. interrogans serovar Icterohaemorrhagiae*, and *L. interrogans serovar Pomona* were inhibited by the addition of 2% glycerol, and partially inhibited with lower concentrations as low as 0.2% [34]. Furthermore, glycerol was found to be toxic to *Streptococcus thermophilus* at 10% and to have no beneficial effect on crystallinity when compared to synthetic cryoprotective polymers [61]. The relative similarity between *Leptospira* and *Borrelia* spirochetes warrants the need for further research in order to study the effect of different cryoprotectant additives on the survival of *B. persica* in frozen cultures.

The results of this study showed that the revival of frozen cultures was surprisingly unaffected by the freezing method used in this study, without any cryoprotectant additive and after direct freezing at −80°C. Isolate aliquots were frozen for 2–3 months before successful defrosting and several passages of the same isolate with freeze–thaw-cycles did not affect the isolates viability. Cryopreservation of *B. persica* and perhaps other RF *Borrelia* spp. isolates with no cryproprotectants may be beneficial for further studies on these pathogens.

## 5. Conclusions

This study describes the first isolation of *B. persica* from a dog. It also indicated that *B. persica* can survive and propagate after direct *in vitro* cultivation in Pettenkofer/LMU Bp medium, without initial isolation in human serum and isolates may proliferate *in-vitro* with stable viability over at least 30 passages. By reaching high cell densities, it is possible to produce a large number of bacteria for further experiments. Furthermore, *B. persica* organisms preserved in frozen cultures may survive and be revived, even after a relatively long period of time, without the use of cryoprotectant additives. Therefore, the cultivation modifications for *B. persica* described here provide an alternative to previously reported *in vitro* methods and to *in vivo* cultivation in laboratory animals.

## Figures and Tables

**Table 1:** Target genes and primers used for PCR to detect and characterize *B. persica*, *E. canis*, *Babesia* spp., and *Hepatozoon* spp.

Pathogen	Target gene	Primers	Amplicon size (bp)	Primer sequence (5′−3′)	Reference
*Borrelia* spp.	*flaB*	Bfpbu	346	GCTGAAGAGCTTGGAATGCAACC	[39]
		Bfpcr		TGATCAGTTATCATTCTAATAGCA	
		BOR1	750	TAATACGTCAGCCATAAATGC	[42]
		BOR2		GCTCTTTGATCAGTTATCATTC	
	*glpQ*	*glpQ*-510f	280	AAAACCCTTTTGGCATAAACAACA	[40]
		*glpQ*-770r		CCAGGGTCCAATTCCGTCAG	
	*16S rRNA*	REC4	515	ATGCTAGAAACTGCATGA	[41]
		REC9		TCGTCTGAGTCCCCATCT	

*E. canis*	*16S rRNA*	E.c. 16S-fwd	123	TCGCTATTAGATGAGCCTACGT	[43]
		E.c. 16S-rev		GAGTCTGGACCGTATCTCAG	

*Babesia* spp. and *Hepatozoon* spp.	*18S rRNA*	Piroplasmid-F	350	CCAGCAGCCGCGGTAATTC	[44]
		Piroplasmid-R		CTTTCGCAGTAGTTYGTCTTTAACAAATC	

## Data Availability

All data generated or analyzed during this study are included in this published article.
